# An evolutionarily stable strategy and the critical point of hog futures trading entities based on replicator dynamic theory: 2006–2015 data for China’s 22 provinces

**DOI:** 10.1371/journal.pone.0172009

**Published:** 2017-02-27

**Authors:** Jinbo Pang, Lingfei Deng, Gangyi Wang

**Affiliations:** College of Economics and Management, Northeast Agricultural University, Harbin, Heilongjiang, China; University of Rijeka, CROATIA

## Abstract

Although frequent fluctuations in domestic hog prices seriously affect the stability and robustness of the hog supply chain, hog futures (an effective hedging instrument) have not been listed in China. To better understand hog futures market hedging, it is important to study the steady state of intersubjective bidding. This paper uses evolutionary game theory to construct a game model between hedgers and speculators in the hog futures market, and replicator dynamic equations are then used to obtain the steady state between the two trading entities. The results show that the steady state is one in which hedgers adopt a “buy” strategy and speculators adopt a “do not speculate” strategy, but this type of extreme steady state is not easily realized. Thus, to explore the rational proportion of hedgers and speculators in the evolutionary stabilization strategy, bidding processes were simulated using weekly average hog prices from 2006 to 2015, such that the conditions under which hedgers and speculators achieve a steady state could be analyzed. This task was performed to achieve the stability critical point, and we show that only when the value of λ is satisfied and the conditions of hog futures price changes and futures price are satisfied can hedgers and speculators achieve a rational proportion and a stable hog futures market. This market can thus provide a valuable reference for the development of the Chinese hog futures market and the formulation and guidance of relevant departmental policies.

## Introduction

Hedgers and speculators comprise the risk allocation framework of futures markets, and these entities represent the main demand for futures contracts and constitute the main trading entities in hog futures bidding. Hedgers trade in the futures market to hedge their investments and to avoid the risks of price fluctuations; speculators seek high profits from speculation [1]. Thus, hedgers tend to transfer risk, whereas speculators are willing to accept price fluctuations to obtain other benefits. The main purpose of listing hog futures is to allow the futures market to perform its risk aversion function and to stabilize hog prices–as well as trading order–and then to reduce losses in hog supply chains. In addition, the price discovery function of the hog futures market can reflect price changes in the spot market for live hogs and other commodities.

As for China, hog futures have not yet been vigorously launched under a comprehensive listing. China is a nation known for hog breeding and hog consumption, and the former is a significant aspect of China's agricultural production and management. However, frequent fluctuations in hog prices have greatly hindered the health and stability of the industry and could seriously affect the entire hog industry [2]. There are both externalities and internal frictions in hog price fluctuations, which not only affect the stability of the domestic hog and agricultural products markets but also impact international hog prices that can lead to instability in foreign hog markets. The experience of foreign countries in hog futures shows that the futures market can fulfill a risk-aversion function, can slow the fluctuations in hog prices, and can promote the development of the hog industry [3–5]. Thus, China must urgently learn from these successful foreign markets and introduce hog futures listing to control irrational trading behavior in the spot hog market.

In recent years, the Dalian Commodity Exchange (DCE) has essentially completed the market research for and design of hog futures contracts and other rules, and the CPC Central Committee and the State Council have clearly stated that measures such as futures trading are necessary to stabilize the hog industry [6,7]. However, China does not yet have a listing for hog futures contracts. In recent years, domestic market research for hog futures has matured, and the preparatory work is nearly finished. The trading process involves four aspects: opening an account and placing the order, bidding, clearing, and settlement [8]. There are many studies of the design aspects and a few studies of bidding. The hog futures market must have a reasonable proportion of the two trading entities to stabilize fluctuations in the futures market price and to be able to minimize risk and perform price discovery functions. Carter and Mohapatra [9] analyzed whether futures prices for nonstorables provide reliable forecasts of cash prices. These authors found that from 1998 to 2004, the hog futures market was an unbiased predictor of cash prices. Elam and Vaught [10] also discussed the risks and benefits of hog futures. These studies all show that the hog futures market must actively cultivate hedgers, guide the appropriate speculators and crack down on excessive speculators, thereby preventing excessive speculation, which leads to market instability.

The domestic agricultural futures market is relatively more mature for purposes of listing hog futures to provide some valuable experience, but it has long been subject to questions regarding (over)speculation. In fact, although it provides a wealth of liquidity for futures market hedgers, China's agricultural futures market has suffered from serious overspeculation and irrational behavior; since its implementation, the futures market has not performed its risk aversion function very well. It is difficult to stabilize price fluctuations in the spot market because of dissipation in the structure of trading entities and because the futures and spot markets are not closely correlated [11]. As domestic hog futures remain unlisted, this paper combines domestic hog market characteristics with the experience of hog futures and domestic agricultural futures from abroad to anticipate future issues and to study the structural relationships of the futures trading entities. However, current studies of the trading activities of futures market hedgers and speculators are limited to theoretical analyses and do not explore the correlation between the two trading entities with the help of models. A group of studies use game models to create trading entities in generator bidding and stock trading, but they do not perform specific game process analysis on futures trading entities [12–14]. The futures bidding process is actually a game process involving hedgers and speculators. Based on the listing needs and characteristics of China’s hog futures combined with relevant domestic and foreign studies and insight derived from a pairwise evolutionary game process involving institutional and individual stock market investors, this paper aims to establish a game model based on replicator dynamic theory to obtain an evolutionarily stable strategy for game players. The purpose of the model is to explore a game relationship between hog futures hedgers and speculators and their stable state. In addition, hog prices were collected for a number of years and incorporated into a game empirical analysis to explore the conditions under which the two trading entities achieve an evolutionarily stable state. Then, certain references are provided for the supervision and control of trading entities once hog futures are listed.

As for evolutionary game theory based on a replicated dynamic model, scholars have focused on various fields and have enriched the research framework of this theory. Cressman noted that evolutionary game theory focuses on the evolution of behavioral types or strategies in biological systems [15]. Moreover, Cressman also noted that Taylor and Jonker describe replication dynamics as the most commonly used dynamic model in an individual evolutionary game theory that equates individual gains directly with their fitness and assumes that their descendants inherit the same strategies [16]. Many scholars have employed evolutionary game and replication dynamic models to study the cooperation strategies, behavior evolution and evolutionary stability strategies of two groups. Sun et al. studied the evolutionary process of group-based emergent events in an uncertain environment and studied the evolutionary process of the strategy selection of strong and weak groups based on evolutionary game theory and finally obtained the behavior evolution regulation of the two groups based on the replication dynamic equation [17]. In addition, many scholars have applied this theory to the strategy selection of different groups in the financial field, which has important reference value for the research of this paper. For example, Friedman noted that the financial markets continue to have new securities and investors enter and exit; on the basis of evolutionary game theory, he found that returns in the financial market model are determined by all price (or revenue) generated by traders’ behavior and behavior selections [18]. Kostanjcar et al. argued that the trading dynamics of buyers and sellers in a financial network are strongly influenced by the (high or low) prices expected by traders and that the dynamic game between buyers and sellers eventually leads to market prices [19]. From the perspective of evolutionary game theory, Zeng and Sun explored the strategy selection issues involved in the collaborative development of a traditional bank and an internet finance company by analyzing the dynamic equation stability solution, and these authors explored its influencing factors using numerical simulation [20]. Peng et al. first inserted the incentive mechanism of compliance innovation developed by regulatory authorities into the analytical framework of evolutionary game theory and then studied the strategy selection issues between financial innovation and incentive regulation in a mutual game before finally analyzing their evolutionary stability strategy using theoretical modeling and numeric simulation [21].

The previous literature mainly involves analyzing the trading behavior and the behavioral strategies of the two groups in the market and the influence of group expectations on their behavior before finally obtaining a dynamic equation stability solution and the stable strategy of the two groups based on evolutionary game theory. Based on this literature, this paper performs a dynamic game analysis on the trading behavior of hedgers and speculators in the hog futures market and determines their stable dynamic solutions. In addition, the empirical analysis is mainly undertaken using real data, looking for the conditions that hedgers and speculators should meet to achieve evolutionary stability strategy and searching to find the rational proportion of the two groups, so as to provide valuable reference for the development of hog futures.

## Methods

Each trading entity in the hog futures market faces a changing economic environment that involves a huge amount of complex and uncertain information that is costly to collect; thus, each trading entity’s information is unique and incongruent with other entities’ information. Therefore, the game played between the trading entities is actually incomplete and asymmetric; their investment behavior is also characterized by bounded rationality, and there is mutual learning and imitation. Each trading entity’s degree of rationality determines the mode and speed of strategic learning and adjustment. Those with a high degree of rationality have strong learning and imitation abilities and can adopt a "fast learning" mode to simulate learning and dynamic adjustment to other trading entities. Those with a low degree of rationality attempt to reach an "evolutionarily stable state" of the biological evolution game by imitating the replicator dynamic mechanism from biological evolution. The purpose of the game analysis in this study is to obtain an evolutionarily stable strategy between hog futures hedgers and speculators using replicator dynamic theory.

### Game model assumptions

The game model in this paper is established based on certain assumptions, which include the following:

Assumption 1: Suppose this game has two players, hog futures hedgers and speculators, both of which engage in hog futures contracts;

Assumption 2: If hog futures hedgers and speculators both have pure strategies, then hedgers’ pure strategy is "buy" or "do not buy" and speculators’ pure strategy is "speculate" or "do not speculate";

Assumption 3: If hedgers do not know speculators’ speculation behavior, and speculators do not know hedgers’ hedging behavior, then the game between the two trading entities is one of incomplete information;

Assumption 4: If hedgers make decisions before speculators, and these trading entities exhibit bounded rationality, (i.e., the balance between the two players is the result of repeated practice, imitation and adjustment), then the game is therefore dynamic; and

Assumption 5: The combined proportion of the two strategies must add up to 1 at any stage of trading.

### Game model construction

The hog futures market includes hedgers, speculators and arbitrageurs, and hedgers and speculators are the main trading entities. Hedger and speculator trading behavior includes both "long sale" trading and "sell short" trading. When futures prices are predicted to rise, traders adopt a "long sale" trading; conversely, when futures prices are predicted to drop, they adopt "sell short" trading. This paper attempts to find an evolutionarily stable strategy for entities that trade hog futures–i.e., hedgers and speculators–based on the assumption that “hog futures hedgers buy a batch of spot hog in the future and are fearful of future spot prices increasing”. Based on this assumption, when hedgers worry about the future spot price increasing, they adopt a "buy" strategy for hog futures based on rational consideration. Some hedgers may adopt measures due to various concerns, such as a "do not buy" strategy for hog futures. Of course, the “do not buy” strategy refers to hog futures, not hog spot holders who worry that the spot price will fall in the future and who therefore adopt a “sell” strategy for hog futures. Hedgers pursue "sell short" trading because they currently have a number of spots and fear that a fall in the future spot price will bring losses. Thus, they adopt a “sell” strategy to make up for the losses. This trading premise is different from the premise of this paper, which is a game process with a similar although opposite trading direction. Speculators’ "long sale" trading and "sell short" trading also reflect their forecasts for the future price. Hedgers’ and speculators’ "long sale" trading and "sell short" trading constitute a hog futures market. To avoid redundancy, this paper performs game analysis and determines an evolutionarily stable strategy for hedgers and speculators using both theoretical and empirical analyses based on the “fear of future spot prices increasing”.

Suppose hog futures hedgers buy a batch of spot hog in the future and fear that the future spot price will increase. If hedgers forecast that the hog futures price will rise based on rational considerations, they will buy hog futures contracts to engage in a hedging strategy. If speculators predict that the hog futures price will rise over the next period of time, they buy hog futures for speculative reasons; if they predict that the hog futures contract price will fall, they do not engage in speculation. Hedging traders essentially buy futures contracts in the same numbers but in the opposite direction as the spot species. Because the futures and the spot prices are affected by the same types of factors, they exhibit the same trend; in the case of a futures trading loss, hedging traders can also seek profits in the spot market to hedge losses. In addition, trading by speculators also involves buying empty or selling short in the futures market. Speculators bear the risk of the futures market; thus, if their market forecast is incorrect and they suffer losses, there is no spot market in which they can retrieve their lost earnings. Thus, futures speculation involves a great deal of speculation, and the futures price risk has a great impact on speculators. The game model is thus constructed as follows:

Suppose hedgers predict that the hog futures price will rise and that this prediction will also involve risk. Therefore, hedgers have two pure strategies, i.e., "buy" or "do not buy"; speculators also have two pure strategies, i.e., "speculate" or "do not speculate".

We define λ as the ultimate probability that the hog futures price will rise and 1-λ as the ultimate probability that the hog futures price will fall.

In the case of an eventual rise in the hog futures price, based on the speculators’ "speculate" strategy, when A_1_ is defined as the earnings from the sale of the hog futures contract when the futures price rises and B_1_ as the loss from paying more for the spot hog when the price rises, the hedgers’ “buy” earning is thus A_1_-B_1_and their “do not buy” loss is B_1_. Based on the speculators’ "do not speculate" strategy, when C_1_ is defined as the earnings from the sale of the hog futures contract when the futures price rises and D_1_ as the loss from paying more for the spot hog when the price rises, the hedgers’ “buy” earning is thus C_1_- D_1_ and the “do not buy” loss is D_1_. As speculators do not face the risk of an increase in the spot price that is based on the hedgers’ “buy” strategy, when E_1_ is defined as the earnings from the sale of the hog futures contract when the futures price rises, the speculators’ "speculate" earning is E_1_, and the "do not speculate" earning is 0. Similarly, based on the hedgers’ “do not buy” strategy, when F_1_ is defined as the earnings from the sale of a hog futures contract when the futures price rises, the speculators’ "speculate" earning is F_1_, and the "do not speculate" earning is 0.

In the case of an eventual drop in the hog futures price, based on the speculators’ "speculate" strategy, when A_2_ is defined as the loss from the sale of the hog futures contract when the futures price drops and B_2_ is defined as the earning from paying less for the spot hog when the price drops, the hedgers’ “buy” loss is thus A_2_-B_2_ and the “do not buy” earning is B_2_. Based on the speculators’ "do not speculate" strategy, when C_2_ is defined as the loss from the sale of the hog futures contract when the futures price drops and D_2_ is defined as the earning from paying less for the spot hog when the price drops, the hedgers’ “buy” loss is thus C_2_- D_2_, and the “do not buy” earning is D_2_. As speculators do not face the risk of spot prices falling that is based on the hedgers’ “buy” strategy, when define E_2_ as the loss from the sale of the hog futures contract when the futures price fall, the speculators’ "speculate" loss is E_2_, and the "do not speculate" loss is 0. Based on the hedgers’ “do not buy” strategy, when F_2_ is defined as the loss from the sale of the hog futures contract when the futures price drops, the speculators’ "speculate" loss is F_2_, and the "do not speculate" loss is 0.

Wherein, A_1_, A_2_, B_1_, B_2_, C_1_, C_2_, D_1_, D_2_, E_1_, E_2_, F_1_, F_2_ are all greater than zero.

According to the aforementioned process, the hedgers’ and speculators’ proceeds matrix can be shown in Table 1.

**Table 1:** Game proceeds matrix for hog futures hedgers and speculators.

		speculators	
		"speculate"	"do not speculate"
hedgers	“buy”	(λ(A_1_-B_1_)-(1-λ)(A_2_-B_2_),λE_1_-(1-λ) E_2_)	(λ(C_1_-D_1_)-(1-λ)(C_2_-D_2_),0)
	“do not buy”	(-λB_1_+(1-λ)B_2_,λF_1_-(1-λ)F_2_)	(-λD_1_+(1-λ)D_2_,0)

Suppose that hog futures hedgers and speculators do not know one another’s strategies and that hedgers make decisions before speculators; in other words, they are participating in a dynamic game characterized by incomplete information. In the auction, hedgers have a ratio x according to which they adopt a "buy" strategy and a ratio 1-x according to which they adopt a "do not buy" strategy; speculators have a ratio y according to which they adopt a "speculate" strategy and a ratio 1-y according to which they adopt a "do not speculate" strategy.

Let us define u_h1_ as the expected proceeds from the hedgers’ "buy" strategy, u_h2_ as the expected proceeds from the hedgers’ "do not buy" strategy, and ${\bar{u}}_{h}$ as the hedgers’ average expected proceeds:

$$u_{h1}=y(\lambda (A_{1}-B_{1})-(1-\lambda )(A_{2}-B_{2}))+(1-y)(\lambda (C_{1}-D_{1})-(1-\lambda )(C_{2}-D_{2}))$$

$$u_{h2}=y(-\lambda B_{1}+(1-\lambda )B_{2})+(1-y)(-\lambda D_{1}+(1-\lambda )D_{2})$$

$$\bar{u_{h}}=xu_{h1}+(1-x)u_{h2}$$

Let us define u_s1_ as the expected proceeds from the speculators’ "speculate" strategy, u_s2_ as the expected proceeds from the speculators’ "do not speculate" strategy, and ${\bar{u}}_{s}$ as the speculators’ average expected proceeds:

$$u_{s1}=x(\lambda E_{1}-(1-\lambda )E_{2})+(1-x)(\lambda F_{1}-(1-\lambda )F_{2})$$

$$u_{s2}=x0+(1-x)0=0$$

$$\bar{u_{s}}=yu_{s1}+(1-y)u_{s2}=yu_{s1}$$

## Results

The core of the bounded rationality game is the dynamic change in the proportion of different strategy types for both players. The specific performance is the dynamic changes in speed and direction, where changes to direction are represented by the speed sign. The imitation speed of hog futures hedgers and speculators determines the strategies that the two trading entities adopt. Generally speaking, the learning and imitation speed of both players is determined by the number and degree of success of mock objects. The former reflects the observation and imitation difficulty level of both players while a given strategy is in effect and can be represented by the proportion of the corresponding strategy type of each game player. The latter reflects the difficulty level of the diversity judgment difficulty and the degree of imitation incentives and can be represented by the magnitude of above-average proceeds of the strategy type of mock objects.

We must study dynamic changes to the strategy of the hedger population and speculator population to obtain the final imitation results of the two players.

Replicator dynamic equations of the hedger population:

$$\begin{array}{l} \frac{dx}{dt}=x[u_{h1}-\bar{u_{h}}]=x(u_{h1}-(xu_{h1}+(1-x)u_{h2}))=x(1-x)(u_{h1}-u_{h2})\\ \;=x(1-x)(y(\lambda A_{1}-(1-\lambda )A_{2})+(1-y)(\lambda C_{1}-(1-\lambda )C_{2}))\\ \;=x(1-x)(\lambda C_{1}-(1-\lambda )C_{2})+y(\lambda A_{1}-(1-\lambda )A_{2}-(\lambda C_{1}-(1-\lambda )C_{2})) \end{array}$$ (1)

Replicator dynamic equations for the speculator population:

$$\begin{array}{l} \frac{dy}{dt}=y[u_{s1}-\bar{u_{s}}]=y(u_{s1}-yu_{s1})=y(1-y)u_{s1}\\ \;=y(1-y)(x(\lambda E_{1}-(1-\lambda )E_{2})+(1-x)(\lambda F_{1}-(1-\lambda )F_{2}))\\ \;=y(1-y)(\lambda F_{1}-(1-\lambda )F_{2})+x(\lambda E_{1}-(1-\lambda )E_{2}-(\lambda F_{1}-(1-\lambda )F_{2})) \end{array}$$ (2)

**Fig 1:**
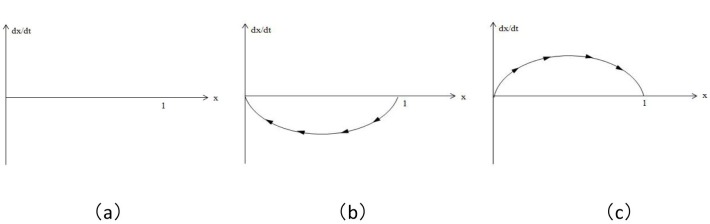
Replicator dynamic phase diagram of the hedger population.

First, the replicator dynamic Eq (1) of the hedger population are analyzed, and the results are as follows:

When y = (-λC_1_+(1-λ)C_2_)/(λA_1_-(1-λ)A_2_-(λC_1_-(1-λ)C_2_)), dx/dt is always 0; i.e., all of x are the evolutionarily stable state, as shown in Fig 1A below.

When y>(-λC_1_+(1-λ)C_2_)/(λA_1_-(1-λ)A_2_-(λC_1_-(1-λ)C_2_)), x* = 0 and x* = 1 are two stable states of replicator dynamic equations, wherein x* = 0 is the evolutionarily stable state, as shown in Fig 1B below.

When y<(-λC_1_+(1-λ)C_2_)/(λA_1_-(1-λ)A_2_-(λC_1_-(1-λ)C_2_)), x* = 0 and x* = 1 are two stable states of replicator dynamic equations, wherein x* = 1 is the evolutionarily stable state, as shown in Fig 1C below.

**Fig 2:**
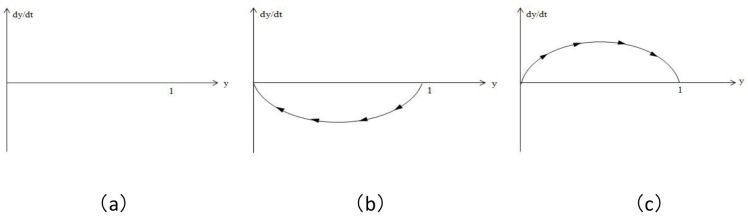
Replicator dynamic phase diagram of the speculator population.

Fig 1 is the replicator dynamic phase diagram of the three cases and steady state of the hedger population:

Then, the replicator dynamic Eq (2) of the speculator population are analyzed, and the results are as follows:

When x = (-λF_1_+(1-λ)F_2_)/(λE_1_-(1-λ)E_2_-(λF_1_-(1-λ)F_2_)), dy/dt is always 0; that is, all of y is the evolutionarily stable state, as shown in Fig 2A below.

When x>(-λF_1_+(1-λ)F_2_)/(λE_1_-(1-λ)E_2_-(λF_1_-(1-λ)F_2_)), y* = 0 and y* = 1 are two stable states of replicator dynamic equations, wherein y* = 0 is the evolutionarily stable state, as shown in Fig 2B below.

When x<(-λF_1_+(1-λ)F_2_)/(λE_1_-(1-λ)E_2_-(λF_1_-(1-λ)F_2_)), y* = 0 and y* = 1 are two stable states of replicator dynamic equations, wherein y* = 1 is the evolutionarily stable state, as shown in Fig 2C below.

**Fig 3:**
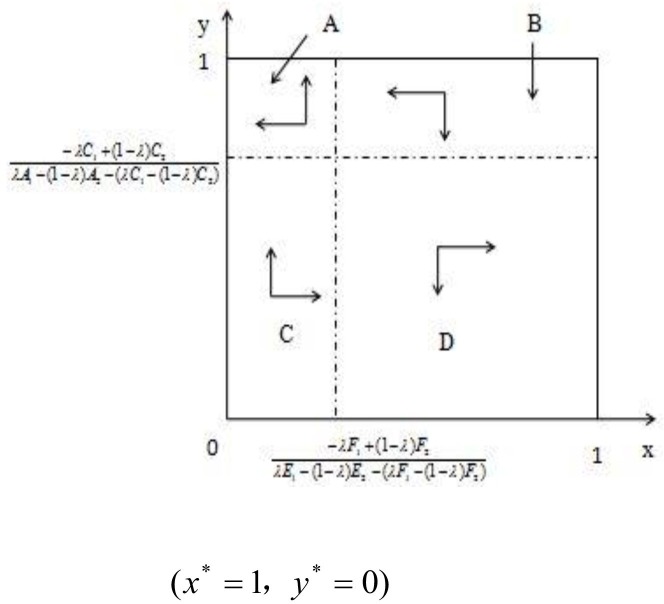
Replicator dynamics and steady state of the hedger and speculator population.

Fig 2 is the replicator dynamic phase diagram of the three cases and steady state of the speculator population:

The replicator dynamic relationship of the hedger population and speculator population proportional change can be shown in a coordinate plane figure. Fig 3 shows the replicator dynamics and steady state of the hedger and speculator populations.

Depending on the direction of the arrow in Fig 3, (x* = 1, y* = 0) and (x* = 0, y* = 1) are the evolutionarily stable strategy in a game that involves hedgers and speculators.

In this replicator dynamics evolutionary game process, when the initial case falls into the A region, it will converge, evolving into stable strategy (x* = 0, y* = 1). In other words, the hedger population adopts a "do not buy" strategy, and the speculator population adopts a "speculate" strategy.

When the initial case falls into the D region, it will converge, evolving into a stable strategy (x* = 1, y* = 0), i.e., the hedger population adopts a "buy" strategy, and the speculator population adopts a "do not speculate" strategy.

When the initial case falls into the B and C regions, it will most likely converge, evolving into stable strategy (x* = 1, y* = 0). In other words, based on long-term learning and strategy adjustment, most of the hedger population will eventually converge and adopt a "buy" strategy, and most of the speculator population will eventually converge and adopt a "do not speculate" strategy, such that the hog futures market can maintain a reasonable proportion of hedgers and speculators, which enforces stability in the hog futures price and performs the function of risk mitigation.

Fig 3 shows that the premise based on which this stable strategy is achieved is as follows:

$$0<\left(\text{-}\lambda F_{1}+\left(1\text{-}\lambda \right)F_{2}\right)/\left(\lambda E_{1}\text{-}\left(1\text{-}\lambda \right)E_{2}\text{-}\left(\lambda F_{1}\text{-}\left(1\text{-}\lambda \right)F_{2}\right)\right)<\left(\text{-}\lambda C_{1}+\left(1\text{-}\lambda \right)C_{2}\right)/\left(\lambda A_{1}\text{-}\left(1\text{-}\lambda \right)A_{2}\text{-}\left(\lambda C_{1}\text{-}\left(1\text{-}\lambda \right)C_{2}\right)\right)<1$$

A stable strategy can be achieved only when the earnings and losses of hedgers and speculators satisfy the above situations, i.e., the hedger population adopts the "buy" strategy, and the speculator population adopts the "do not speculate" strategy.

## Discussion

**Fig 4:**
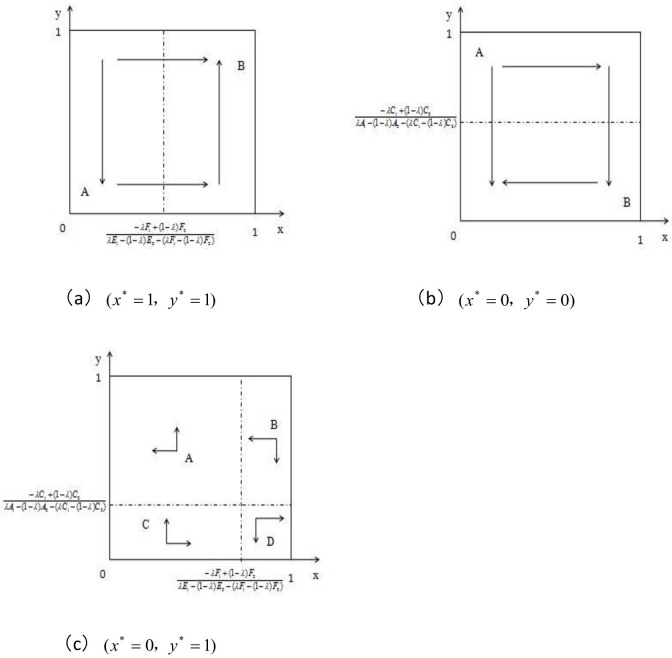
Replicator dynamics and steady state of the hedger and speculator population.

In this bidding game, in addition to the ideal steady state in which hedgers adopt the "buy" strategy and speculators adopt the "do not speculate" strategy, there are also several other stable states. These specific stable states are determined by the relationship between (-λC_1_+(1-λ)C_2_)/(λA_1_-(1-λ)A_2_-(λC_1_-(1-λ)C_2_)) and(-λF_1_+(1-λ)F_2_)/(λE_1_-(1-λ)E_2_-(λF_1_-(1-λ)F_2_)), which are defined as M and N. M and N can be regarded as the critical points at which hog futures hedgers and speculators realize that only when the value of λ, the hog spot price and hog futures prices meet the threshold under the stable strategy can the trading entities achieve the evolutionarily stable strategy. Several main stable states are as follows:

1. When M<0, 0<N<1, the proportional change in the replicator dynamic relationship of the hedger population and the speculator population can be shown in a coordinate plane figure as follows:

As shown by Fig 4A, (x* = 1, y* = 1) is the evolutionarily stable state of the game process, and if it is repeated over a long period during the game, the proportion of the hedger population adopting a "buy" strategy will gradually increase to 1, i.e., almost all the hedgers will begin to realize that a "buy" strategy is the most desirable strategy of self-interest. As long as in the initial stage of the game there are speculators adopting the "speculate" strategy, an increasing number of speculators will follow this investment behavior, and the proportion of speculators adopting the "speculate" strategy will gradually increase until it reaches 1. Trading in the hedger population and speculator population under the steady state will be extremely active, which may lead to excessive speculation in the hog futures market and aggravate fluctuation in the hog spot market price, leading to instability in the hog futures market and the spot market.

2. When N<0, 0<M<1, the replicator dynamic relationship of the proportional change in the hedger population and speculator population can be shown in the coordinates plane figure as follows:

As shown by Fig 4B, (x* = 0, y* = 0) is the evolutionarily stable state of the game process; if this state is repeated over a long period, the proportion of the hedger population adopting a "buy" strategy will gradually decrease to 0. Thus, almost all the hedgers will begin to realize that a "do not buy" strategy is the most desirable strategy of self-interest. In addition, as long as in the initial stage of the game there are speculators adopting the "do not speculate" strategy, there will be an increasing number of speculators who follow this investment behavior, and the proportion of the speculator population who adopt the "speculate" strategy will gradually decrease until it reaches 0; in other words, there will be a growing number of speculators who adopt a "do not speculate" strategy. The hedger population and speculator population adopt negative strategies under the steady state, which can seriously affect hog futures’ liquidity and lead to an extremely inactive market. Finally, the hog futures market cannot fulfill the risk-averse function.

3. When0<M<N<1, the replicator dynamic relationship of the proportional change in the hedger population and speculator population can be shown in the coordinate plane figure as follows:

As shown in Fig 4C, (x* = 0, y* = 1) and (x* = 1, y* = 0) are the evolutionarily stable states of the game process. If this state is repeated over a long period–given the long-term learning and strategy adjustment–most of the hedger population will eventually converge and adopt a "do not buy" strategy, and most of the speculator population will eventually converge and adopt the "speculate" strategy, i.e., converge and evolve into stable state (x* = 0, y* = 1). Under this stable state, hedger population trading is extremely inactive, whereas speculator trading is extremely active, which leads to excessive speculation and price fluctuations in the hog futures and hog spot markets.

### Empirical analysis

The bidding game model analysis of the hog futures intersubjective game steady state is too abstract. To concretely portray the game process, this paper collected real data on China’s hog prices, simulated a real game state that involved hog futures intersubjective, and then sought to determine an evolutionarily stable strategy and the critical point between hedgers and speculators.

### Data collection and research time interval selection

To observe and analyze fluctuations in hog prices and to find the appropriate time interval for intersubjective empirical analysis, we collected the weekly average hog price of 22 provinces for the 2006–2015 period.

**Fig 5:**
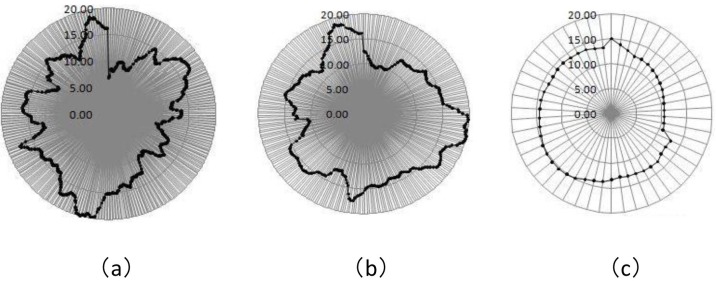
Fluctuations in average weekly hog prices for different time periods.

Fig 5 shows the weekly average hog price fluctuation at different time periods, where Fig 5A is a radar graph depicting the weekly average fluctuation in hog prices from 2006/7/14 to 2015/11/20, Fig 5B is a radar graph depicting the weekly average fluctuation in hog prices from 2010/1/8 to 2015/11/20, and Fig 5C is a radar graph depicting the weekly average fluctuation in hog prices from 2014/1/3 to 2014/12/26. As shown by these figures, Fig 5C has the lowest price fluctuation, as the prices basically fall within the range of 10–15 yuan/kg and the decline in price and the extent of the increase are small. Fig 5A and 5B have higher price fluctuations than Fig 5C, and the prices basically fall within the range of 5–20 yuan/kg. Fig 5A has higher and more frequent price fluctuations than Fig 5B, which reflects a more obvious cobweb phenomenon, and the data also reflect the hog prices fluctuation rule. Therefore, hog futures price data spanning a decade are more scientifically rigorous, and the results are more representative.

**Fig 6:**
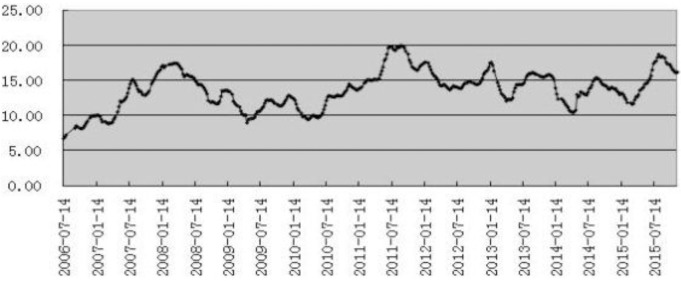
Fluctuations in the average weekly hog price from 2006 to 2015.

Fig 6 reflects the trends and fluctuations in the weekly average hog price of China’s 22 provinces. As shown in Fig 6, the sharp fluctuation interval can be divided into five stages in this decade. Thus, there was an overall upward trend from 2006/7/14 to 2008/3/21, from 6.76 yuan/kg to 17.45 yuan/kg; there was an overall downward trend from 2008/3/28 to 2009/4/30, from 17.40 yuan/kg to 8.98 yuan/kg; there was an overall upward trend from 2009/5/8 to 2011/9/9, from 9.45 yuan/kg to 19.92 yuan/kg; there was an overall downward trend from 2011/9/16 to 2014/4/18, from 19.78 yuan/kg to 10.45 yuan/kg; and there was an overall upward trend from 2014/4/25 to 2015/11/20, from 10.55 yuan/kg to 16.18 yuan/kg.

**Fig 7:**
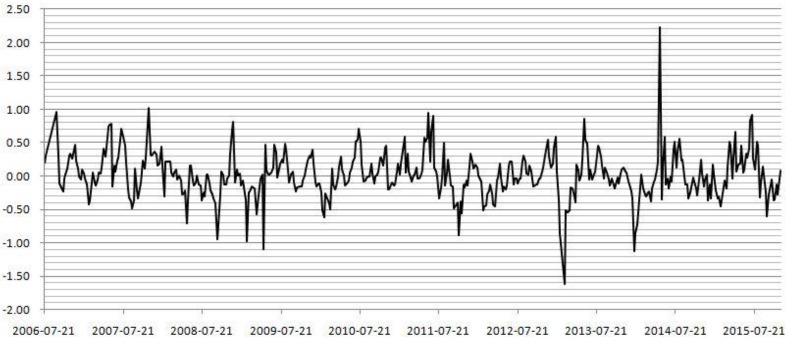
Difference sequences of weekly average hog price from 2006 to 2015.

Fig 7 is a 2006–2015 hog price difference sequence diagram in which each sequence is the difference between the current week’s average price and the previous week’s average price. As shown by Fig 7, the periodic fluctuation in hog prices alternatively shows ups and downs, and the fluctuation ranges of the price difference basically falls at [–1,1]; notably, there are also few high fluctuation outliers, which may be the result of a number of unexpected factors. For example, the weekly average hog price rose 2.23 yuan in 2014/5/9 relative to the previous week, and this round is considered an irrational rise in price. In addition to the periodic hog source tension and holiday effect, there may be other non-market factors affecting prices (e.g., May 1 is International Labor Day).

As shown by the weekly average hog price trends and the difference sequences, China’s hog price fluctuates frequently over the decade, and the fluctuation exhibits large amplitude and high frequency characteristics, which is not conducive to stability. Foreign countries pursue economic freedom, which is beneficial for a country's economic development; thus, fluctuations in hog prices are not subject to macro-level control. The United States, Germany and other countries try to stabilize hog prices with the help of hog futures and the futures market price discovery function. However, due to China's national conditions, governmental macro-level control not only affects changes in prices in the market economy but also has an even more profound impact on the quality of life of urban and rural residents [22,23]. China’s hog prices remain in a state of flux [24–26], and hog futures contracts are an effective tool to alleviate this fluctuation. Implementing these contracts is important for China.

Based on the above analysis, we select a rise interval and a fall interval to perform an empirical analysis of the hog futures trading entities bidding game.

### Simulation process of hog futures trading entities bidding game

The simulation process involves an empirical analysis using specific data on the basis of the theoretical game results followed by verification of the theoretical game results and an attempt to understand the hog market realities reflected in the conclusions. We divided the simulation process into three parts: first, the ultimate hog futures contract probability of rising λ is solved; second, the expected price increase interval and the expected price decrease interval within the 2006–2015 average weekly hog prices are selected; and finally, the stable strategy and the conditions required to meet this strategy are analyzed.

1. Solving the value of λ. λ indicates the probability of an eventual rise in the price of a hog futures contract. Because the hog price and the hog futures price change in the same direction and the change in direction and degree of variation are affected by many of the same factors, this paper selects the relevant data for hog prices to solve the value of λ. The value of λ changes with changes in the hog price, and there are many factors that affect hog price, such as production costs, prices of alternatives, resident income, sudden outbreaks, food safety incidents, the economic environment and major policy changes. When factors experience large changes, there is fluctuation in hog prices, resulting in price outliers. To ensure the authenticity of the λ solution results, we have not excluded outliers. To synthetically consider both change frequency and the extent of change in prices, the solution process is subdivided into the following sections (data are from the 2006–2015 average weekly hog price in China’s 22 provinces):

(1) Solving the increment between the current week average price and the previous week average price. The results show that the interval of negative increments is [-1.62, -0.01], for a total of 228, which account for 0.5 of the fluctuation; the interval of positive increments is [0.01, 2.23], for a total of 220, which account for 0.48 of the fluctuation. Because so few prices have no changes, we ignored these data in the following solution process; thus, fluctuation only involves rising and falling prices.

(2) Solving the ratio of each negative increment with the minimum negative increment, the negative increment is divided into three intervals, i.e., [-1.62, -1.00], [-1.00, -0.50], [-0.50–0.01]. The negative increment interval segment is based on an equidistant segments standard, as only three negative increments are less than -1.00, i.e., -1.10, -1.12, and -1.62, which cannot achieve equidistant segments. Therefore, [-1.62, -1.00] is a segment interval, and the negative increment greater than -1.00 is based on the equidistant segments standard. Then, each ratio average within each interval is solved, 0.7901, 0.4133, and 0.1082, respectively, and the final probability of the negative increment is obtained as follows:

0.50*(0.7901+0.4133+0.1082)=0.6558

(3) To solve the ratio of each positive increment with the maximum positive increment, the positive increment is divided into three intervals, i.e., [0.01, 0.50], [0.50, 1.00], and [1.00, 2.23]. The positive increment interval segment is based on the equidistant segments standard, as only two positive increments are greater than 1.00, i.e., 1.02 and 2.23, which cannot achieve equidistant segments. Therefore, [1.00, 2.23] is a segment interval, and the positive increment less than -1.00 is based on the equidistant segments standard. Then, each ratio average within each interval is solved, 0.7287, 0.2962, and 0.0805, respectively, and the final probability of the positive increment is obtained as follows:

0.48*(0.7287+0.2962+0.0805)=0.5333

(4) The final value of λ is solved as follows:

λ=0.5333/(0.5333+0.6558)=0.4485

1-λ=0.6558/(0.5333+0.6558)=0.5515

Therefore, the final values of λ and 1-λ are 0.4485 and 0.5515, which synthetically consider both the change frequency and the extent of change in price.

The same method is used to solve the probability of a rise in 2010–2015 hog prices, i.e., the value of λ is obtained. There are a total of 149 negative increments, accounting for 0.51 of fluctuation, and a total of 138 positive increments, accounting for 0.49 of fluctuation, which are similar to the ten-year ratio. The final values of λ and 1-λ are 0.4838 and 0.5162, respectively. According to the value of λ and 1-λ from the ten-year and six-year periods, the gap between the probability of a rise in the hog price and a fall in the hog price is small. While the rise in probability is a little less than the fall in probability, the rise is approximately 0.45, and the fall is approximately 0.55. For hog futures hedgers and speculators in China, this probability is conducive to their anticipation of the prices of hog and hog futures contracts and is the basis for formulating rational trading decisions.

2. Selecting the expected price change interval. Futures trading, i.e., a forward trade of 3 months, 6 months, 1 year, etc., arranges a physical delivery in a fixed month in accordance with the time, place and quantity of the underlying commodity. Wheat futures contracts in the CME, for example, have delivery months of July, September and December of that year and March and May of the next year. Moreover, futures traders can select the delivery time based on their trading needs. If futures traders purchase a contract with a delivery month of July, hedgers should make a physical delivery in July, and speculators can close a position at a certain time before July to settle the trading. To simplify the game analysis process, we assume that both hedgers and speculators trade when the futures contract expires and that the trading deadline is six months. On this basis, the selected interval of an expected rise in price is from 2007/10/12 to 2008/04/12, where hog prices rise from 12.86 yuan/kg to 17.34 yuan/kg, and the selected interval of an expected fall in prices is from 2013/08/28 to 2014/02/28, where hog prices fall from 16.05 yuan/kg to 11.92 yuan/kg.

3. Solving the stable state and the critical points. Because hog futures are not listed in China, we assumed the value of hog futures in solving the stable state. The spot price and futures price exhibit correlation [27–29]; thus, when prices trend upward, the futures price is higher than the spot price, and when prices tend to fall, the futures price is lower than the spot price. If the hog price and price change trends are considered synthetically, the hog futures contract price is assumed to be the following: hog contract prices in the interval of an expected rise in prices are set to rise from 12 yuan/kg to 18 yuan/kg, and hog contract prices in the interval of an expected fall in prices are set to fall from 15 yuan/kg to 10 yuan/kg. The trading activity of hedgers and speculators will affect the futures contracts price; therefore, when speculators select “speculate”, the futures market is active, the contract value is higher than that of “do not speculate”, the hedger “buy” strategy price is higher than that of “do not buy”, and the “speculate” price is higher than that of “buy”. Thus, hog futures prices are further assumed in Table 2 below:

**Table 2:** Assumptions of hog futures contract price unit: yuan/kg.

	"speculate"	"do not speculate"	“buy”	“do not buy”
up interval	12–18	12.3–17.7	12.1–17.9	12.2–17.8
down interval	15–10	12.7–10.4	14.9–10	14.8–10.3

The hog futures hedger and speculator benefit matrix is shown in Table 3.

**Table 3:** Proceeds matrix for hog futures hedgers and speculators.

		speculators	
		"speculate"	"do not speculate"
hedgers	“buy”	(0.2019,-0.1011)	(0.3189,0)
	“do not buy”	(0.2684,0.0299)	(0.2684,0)

Critical points M and N are obtained as follows:

$$M=(\text{-}\lambda C_{1}+(1\text{-}\lambda )C_{2})/(\lambda A_{1}\text{-}(1\text{-}\lambda )A_{2}\text{-}(\lambda C_{1}\text{-}(1\text{-}\lambda )C_{2}))=0.4316$$

$$N=(\text{-}\lambda F_{1}+(1\text{-}\lambda )F_{2})/(\lambda E_{1}\text{-}(1\text{-}\lambda )E_{2}\text{-}(\lambda F_{1}\text{-}(1\text{-}\lambda )F_{2}))=0.2282$$

The relationship 0<N<M<1 is found between critical point M and N; within the selected expected price interval, the evolutionarily stable strategy of hog futures hedgers and speculators is (x* = 1, y* = 0), and the critical points M and N are 0.4316 and 0.2282, respectively. Where the previously described value of λ is satisfied, and the conditions of hog futures price changes and futures price assumptions are satisfied, hedgers and speculators can achieve an evolutionarily stable strategy (x* = 1, y* = 0). Under this steady state, hedgers adopt a "buy" strategy, and speculators adopt a "do not speculate" strategy. Moreover, under this state, the majority of trading entities will tend to choose this strategy, forming the optimal ratio of hedgers and speculators.

## Conclusion

China’s hog market prices have been showing abnormal fluctuation, and hog futures have not been listed; thus, governmental macro-level control has been relied on to stabilize hog price fluctuations and prevent hog market failures. Agricultural policy and hog policy have played a role throughout the development of the hog industry, and macro-level control is thus an important factor in the industry. The purpose of these policies can be broadly divided into two categories, i.e., the prevention of excessive rises and excessive drops in hog prices. However, the current regulatory effect of a series of macro-level control measures has been questioned by many people, and these measures have not achieved the goal of stabilizing market supply and safeguarding consumer demand. Controlling hog price fluctuations was unsuccessful and even resulted in a market rebound due to the excessive nature of the measure, which was then followed by an exacerbation of hog price fluctuations. In advancing the process of agricultural modernization, the United States mainly relies on market regulation based on the voluntary participation of farmers in implementing indirect controls, which leads to a win-win situation. The United States attempted to exploit the price discovery and risk aversion function of markets and then used the free market to control hog prices; this approach also contributed to the scale and standardization of the hog industry in the US. China should also exploit the autoregulation function of markets to stabilize fluctuations in the hog market. Thus, hog futures should be used as a tool for market regulation, reducing the government's macro-level control of the market and leading to real market liberalization. The conclusions are as follows:

1. By analyzing the development of domestic and foreign hog and other commodity futures markets, we confirm that the main purpose of hog futures is to allow hedging of the spot market, thus stabilizing hog prices, creating steady income streams for farmers and providing hedging instruments for hog spot enterprises. Although hog futures speculation can play a positive role in futures trading, speculation risk can lead to instability in the hog futures market. Thus, the trading entities must reach an evolutionarily stable strategy through game theory under certain conditions to promote the stability and sustainability of the market.
2. We use replicator dynamic equations to explore the evolutionarily stable strategy of hog futures trading entities based on evolutionary game theory. By analyzing the behavior of hog futures trading entities (hedgers and speculators), we find that there is more than one evolutionarily stable state of these two trading entities, although there is only one strategy that is conducive to market stability: exploiting the risk aversion function of the futures market and then pursuing the original intention of hog futures listing in which hedgers adopt a "buy" strategy and speculators adopt a "do not speculate" strategy.
3. In actual trading, it is difficult to reach the extremely stable state that we find from constructing and analyzing the game model. In addition, too many hedgers will reduce liquidity; an appropriate amount of speculators is required to expand market capacity and the trading size of hog futures in addition to maintaining a relatively stable state between hog futures markets and trading entities. Thus, the stable state in the actual hog futures market is a situation closer to this extreme stable state, and there will thus be many hedgers adopting a "buy" strategy and a small amount of speculators adopting a “do not speculate” strategy in the hog futures market. In this manner, the optimal ratio of hedgers and speculators is reached, the hog futures market is stable, and the best conditions of market activity are maintained.
4. To find the optimal proportion discussed above by collecting the actual data of the domestic hog market, we analyze the conditions that must be met for trading entities to reach a stable strategy through empirical analysis. The empirical results show that only when the above-described value of λ is satisfied and hog futures price conditions and futures price assumptions are changed, i.e., when the critical point under a stable-state strategy is achieved, can hedgers and speculators reach this stable state. When these conditions are met, the optimal proportion of hedgers and speculators can be obtained.
5. Based on the above conclusions, the relevant departments can adopt policies or appropriate guidance for the hog futures market in light of China's national conditions and market rules. The relevant departments should implement a hog futures market based on price trends and fluctuations in the hog spot market and based on the expectations of the market to actively cultivate hedgers, to actively guide the appropriate speculators, and to punish excessive speculators, thus avoiding the price risks inherent in the hog spot market.

## Supporting information

S1 File Fig 5 in S1 File. (XLS)

S2 File Fig 6 in S2 File. (XLS)

S3 File Fig 7 in S3 File. (XLS)

S4 File Table 3 in S4 File. (XLS)
