# Correction: An evolutionarily stable strategy and the critical point of hog futures trading entities based on replicator dynamic theory: 2006-2015 data for China's 22 provinces

**DOI:** 10.1371/journal.pone.0182702

**Published:** 2017-08-01

**Authors:** Jin-Bo Pang, Ling-Fei Deng, Gang-Yi Wang

All of the authors’ names are spelled incorrectly. The correct author list is: Jin-Bo Pang, Ling-Fei Deng, Gang-Yi Wang. The correct citation is: Pang J-B, Deng L-F, Wang G-Y (2017) An evolutionarily stable strategy and the critical point of hog futures trading entities based on replicator dynamic theory: 2006–2015 data for China’s 22 provinces. PLoS ONE 12(2): e0172009. https://doi.org/10.1371/journal.pone.0172009
